# Passive immune therapy and other immunomodulatory agents for the treatment of severe influenza: Systematic review and meta‐analysis

**DOI:** 10.1111/irv.12699

**Published:** 2019-11-16

**Authors:** Vanessa W. Lim, Lorainne Tudor Car, Yee‐Sin Leo, Mark I‐Cheng Chen, Barnaby Young

**Affiliations:** ^1^ National Centre for infectious Diseases Singapore City Singapore; ^2^ Lee Kong Chian School of Medicine Nanyang Technological University Singapore City Singapore; ^3^ School of Public Health Imperial College London London UK; ^4^ Saw Swee Hock School of Public Health National University of Singapore Singapore City Singapore; ^5^ Yong Loo Lin School of Medicine National University of Singapore Singapore City Singapore

**Keywords:** adjunctive therapies, influenza, mortality, passive immune therapy

## Abstract

**Background:**

A range of immunomodulatory therapies have been proposed as adjuncts to conventional antivirals to suppress harmful inflammation during severe influenza infection. We conducted a systematic review to assess available data of the effect of adjunctive non‐corticosteroid immunomodulatory therapy and potential adverse effects.

**Method:**

We searched MEDLINE, Embase, Web of Science and clinical trial databases for published and unpublished studies, and screened the references of included articles. We included RCTs, quasi‐RCTs and observational studies of virologically confirmed influenza infections in hospitalised patients. We did not restrict studies by language of publication, influenza type/subtype or age of participants. Where possible, we pooled estimates of effect using random‐effects meta‐analysis models.

**Results:**

We identified 11 eligible studies for inclusion: five studies (4 RCTs and 1 observational; 693 individuals) of passive immune therapy; four studies (3 RCTs and 1 observational; 1120 individuals) of macrolides and/or non‐steroidal anti‐inflammatory drugs (NSAIDs), one RCT of mTOR inhibitors (38 individuals), and one RCT of statin therapy (116 individuals). Meta‐analysis of RCTs of passive immune therapy indicated no significant reduction in mortality (OR 0.84, 0.37‐1.90), but better clinical outcomes at Day 7 (OR 1.42, 1.05‐1.92). There was a significant reduction in mortality associated with macrolides and/or NSAIDs (OR 0.28; 0.10‐0.77).

**Conclusions:**

Passive immune therapy is unlikely to offer substantial mortality benefit in treatment of severe seasonal influenza, but may improve clinical outcomes. The effect of other immunomodulatory agents is uncertain, but promising. There is a need for high‐quality RCTs with sufficient statistical power to address this evidence gap.

## 1 INTRODUCTION

Seasonal influenza is a common viral infection of the respiratory tract. It is estimated to cause more than a billion infections annually, with three to five million severe illnesses and 250 000‐650 000 deaths.^1^, ^2^ The highest mortality rates have been reported in adults aged over 75 years, children younger than 5 years and residents of sub‐Saharan Africa or South‐East Asia.

Recommended antiviral treatments of severe seasonal influenza are currently limited to the neuraminidase inhibitors (NAIs).^3^, ^4^ While effective at shortening the duration of influenza symptoms when administered early in the course of infection, debate continues as to the extent NAIs are able to prevent progression to severe infection, the development of complications in hospitalised individuals, or reduce mortality.^5^, ^6^

An effective immune response to the influenza virus following infection is necessary for viral clearance and recovery from infection. Viral shedding is prolonged in immunocompromised patients with influenza, associated with an increased risk of emergent NAI resistance, and secondary bacterial infections.^7^, ^8^, ^9^

But in a delicate balance, this immune response to an infection can also be harmful to the host. For example, an excessively pro‐inflammatory cytokine and chemokine environment has been cited as the key explanation for the severity of human infections with highly pathogenic avian influenza, and the 1918 H1N1 “Spanish flu” pandemic.^10^ This “cytokine storm” can rapidly result in multi‐organ dysfunction and acute respiratory distress syndrome (ARDS). Similarly, in seasonal influenza damage to the airways and alveolae is mediated both by viral replication and by the innate immune response.^11^

A range of immunomodulators for severe influenza have been proposed,^12^, ^13^ but certainty as to their relative benefits and harms is lacking. Corticosteroid therapy, for example, is widely prescribed as part of the standard of care for treatment of influenza complications such as the treatment of exacerbations of chronic obstructive pulmonary disease (COPD) and asthma.^14^, ^15^

A Cochrane review in 2017 found moderate‐quality evidence that corticosteroids also reduce mortality when used in severe community‐acquired pneumonia (relative risk [RR] 0.58; 95% CI: 0.40‐0.84).^16^ Conversely, however, in the context of severe influenza, an updated Cochrane meta‐analysis published in 2019 concluded that corticosteroid therapy was associated with increased mortality (odds ratio [OR] = 3.90; 95% CI: 2.31‐6.60; *I* ^2^ = 68%; 15 studies).^17^ This result must be interpreted with caution as it was mainly derived from observational studies and residual bias is likely to persist as patients with more severe influenza are more likely to receive corticosteroids.

The recent 2018 Infectious Diseases Society of America (IDSA) seasonal influenza guidelines do not recommend any immunomodulatory therapies as adjunctive treatments.^3^ This systematic review focuses on immunomodulatory agents other than corticosteroids for the treatment of severe influenza. Three systematic reviews of passive immune therapy (convalescent plasma/serum or intravenous immunoglobulin) for the adjunctive care of severe influenza were previously published.^18^, ^19^, ^20^ These reviews, however, included only data from non‐randomised studies and historical reports from the 1918 Spanish influenza pandemic which are of uncertain relevance today. A number of randomised controlled studies of passive immune therapy have been published since.

This systematic review was commissioned by the World Health Organization (WHO) to inform the development of clinical practice guidelines for severe influenza. It aims to provide a comprehensive and up‐to‐date assessment of the available data investigating the clinical effectiveness and safety of non‐corticosteroid immunomodulatory therapy adjunctive to conventional antiviral medication for the treatment of severe influenza.

## 2 METHODS

This systematic review and meta‐analysis was conducted in accordance with the PRISMA Statement 2009.^21^ A search construct was developed and applied to the Cochrane Central Register of Controlled Trials (CENTRAL), MEDLINE and EMBASE databases of published literature (Appendix for the MEDLINE search construct and full inclusion/exclusion criteria).

Only studies conducted in humans with a virologically confirmed influenza infection (seasonal or zoonotic) were included, but without restriction on age or sex. There was no restriction on date or language of publication. We included randomised trials, quasi‐experimental and observational studies published in academic, peer‐reviewed literature. Population studies and studies with fewer than 10 participants were excluded. For studies with an observational design, only studies that attempted to adjust for differences between groups in disease severity and/or propensity to receive the immunomodulatory therapy were included.

Clinicaltrials.gov and the WHOs International Clinical Trials Registry Platform (ICTRP) were also searched for ongoing clinical trials, and data from these studies were included if the study was completed and results were available from online sources such as the clinical study report. Web of Science was used for citation searching by collating the bibliographies and citations of included studies to identify additional studies which may be eligible.

We included studies of any of passive immune therapy, macrolides, mTOR inhibitors, non‐steroid anti‐inflammatory drugs (NSAIDs) or statins. Comparator groups were those who received antiviral therapy or supportive care alone.

Our primary outcome of interest was mortality, and secondary outcomes were severity of illness (eg requiring admission to intensive care and/or mechanical ventilation), duration of hospital admission, serious adverse events, duration of viral shedding and emergence of resistance.

Studies were selected in two stages: first review of study title and abstract, followed by analysis of the full text of the article. Each study was independently reviewed by two authors, and disagreements were resolved by discussion (VL, BY). Data from studies to be included in the review were then independently extracted by these two review authors using a standardised data collection form developed and piloted for this systematic review (Appendix ).

Two review authors independently assessed the methodological quality of included studies (VL, BY). RCTs were assessed using the Cochrane Risk of Bias (RoB 2.0) tool, while non‐randomised studies were assessed using a Newcastle‐Ottawa Scale (NOS) modified for the purposes of this review (Appendix ).^22^, ^23^

Mortality data from individual studies were tabulated and odds ratios (OR) with 95% confidence intervals (CI) calculated using Review Manager 5.3.^24^ For RCTs, data were analysed on an intention‐to‐treat (ITT) basis. No form of data imputation was used for participants with missing outcome data. Outcome measures that have been adjusted for confounding, such as ORs or hazard ratios (HRs) with 95% CIs, were also extracted. Where multiple adjusted analyses were presented, the results from the most complete model were collected. Ordinal logistic regression and additional statistical tests were performed using R version 3.6.1 as required.

The *I* ^2^ statistic was used to assess heterogeneity across experimental and observational studies. An *I* ^2^ value >75% was inferred to reflect substantial heterogeneity between the findings from the studies.

Outcome data from observational studies were aggregated using a random‐effects meta‐analysis model to pool data to reflect expected differences in the measured effectiveness of adjunctive therapies—due to differing patient characteristics, interventions and outcome definitions. Data from different therapies and study designs (experimental vs observational) were aggregated separately and combined when considered appropriate.

The five Grading of Recommendations, Assessment, Development and Evaluation (GRADE) considerations (study limitations, consistency of effect, imprecision, indirectness and publication bias) were used to assess the quality of evidence from the studies that contribute data to the meta‐analyses for the pre‐specified outcomes. Reasons for the decision to downgrade or upgrade the quality of studies were provided.

## 3 RESULTS

**Figure 1:**
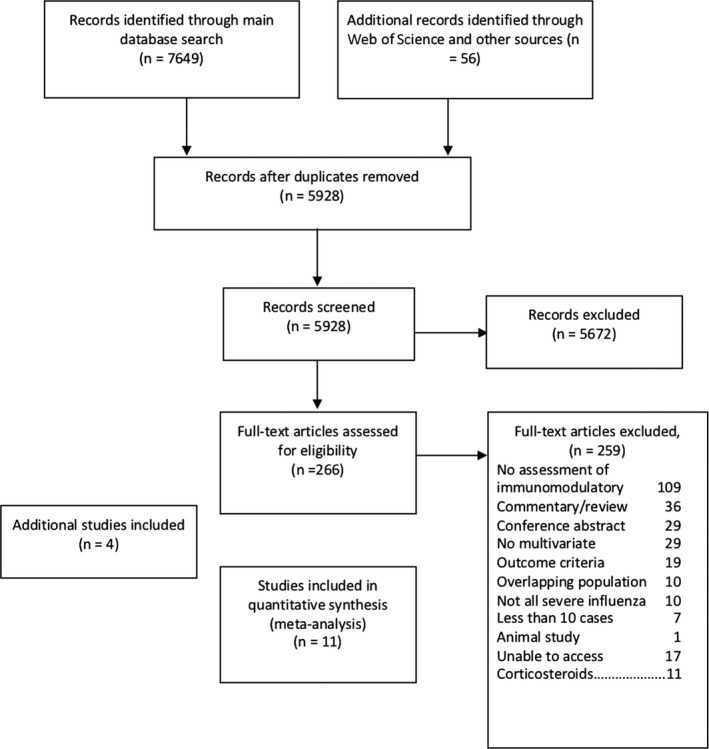
PRISMA flow chart

The search strategy was implemented on 25 January 2019, and identified 5928 articles after removal of duplicates (Figure 1). An additional 56 articles were identified through reference tracking (though none of these met eligibility criteria to be included in the full‐text review). The full text of 266 articles was scrutinised, and seven were initially identified for inclusion in the systematic review. Four additional completed studies were identified from clinicaltrials.gov, which at the time of review were unpublished, but with data available from various online sources including the clinical study record. Two of these studies have since been published.^26^, ^27^  All studies had virologically confirmed influenza through a combination of either laboratory polymerase chain reaction or rapid antigen test.

The main reason for article exclusion was no reported assessment of the effect of adjunctive immunomodulatory treatment on patient outcomes. Seventeen articles were identified as potentially eligible, but we were unable to locate full‐text copies of the article. Fourteen of these were not published in English, and all were published prior to 2011.

Five studies on passive immune therapy, two of macrolides, one of NSAIDs, one of NSAIDs in combination with macrolides, one of mTOR inhibitors, and one of statins were identified. The study design, intervention, participants and outcome characteristics are summarised in Table 1.

**Table 1:** Summary of study design, inclusion/exclusion criteria and participants of studies of adjunctive passive immune therapy for influenza

Reference	Study design	Country (no sites), y	Influenza type	Setting	No participants (exp: cont %)	Sex (male)	Age, y (median, IQR)	Major inclusion criteria	Study primary endpoint	Mortality definition	Mortality (control group)
Passive immune therapy											
Hung et al^29^	Prospective cohort	Hong Kong (7), 2009‐10	Pandemic H1N1	ICU	93 (22:78)	68.8%	Exp: 48 (37‐56); Cont: 54 (43‐62)	≥18 y; deterioration despite antivirals	Not stated	In‐hospital	54.8%
Hung et al^25^	Placebo‐controlled RCT	Hong Kong (1), 2010‐11	Pandemic H1N1	ICU	35 (49:51)	55.9%	Exp: 43 (37‐56); Cont 41‐59)	≥18 y; severe CAP; deterioration despite antivirals	Mortality	21‐d mortality	23.5%
Beigel et al^28^	Open‐label RCT	US (29), 2011‐15	Seasonal (A + B)	Hospital	87 (48:52)	48.0%	Exp: 50 (38‐66); Cont: 57 (39‐71)	Hypoxia or tachypnoea. Exclusion: suspicion that main illness not due to flu	Time to normalisation of patients’ respiratory status	In‐hospital	11.1%
Davey et al 26	Blinded, placebo‐controlled RCT	US, Thailand, UK, Spain + 5 others (33), 2013‐18	Seasonal (A + B)	Hospital	329 (51:49)	45.5%	Exp: 55 (41‐68); Cont: 57 (48‐68)	Illness onset ≤7 d; New score ≥2	Outcome at Day 7 (ordinal scale)	7‐day mortality	1.3%
Beigel et al^27^	Blinded, placebo‐controlled RCT	US (30), 2015‐19	Seasonal (A)	Hospital	138 (66;34)	51.4%	Exp: 58(47‐69); 63 (44‐69)	Illness onset ≤7 d; New score ≥3	Outcome at Day 7 (ordinal scale)	28‐day mortality	4.3%
Macrolide											
Martin‐Loeches et al^30^	Prospective cohort	Spain (148), 2009‐11	Pandemic H1N1	ICU	733 (26:74)	60.0%	Exp: 44 ± 14.0; Cont: 46 ± 13.9	≥15 y; Primary viral pneumonia	Mortality	In ICU	28.1%
Lee et al^31^	Open‐label RCT	Hong Kong (3), 2013‐16	Seasonal (A + B)	Hospital	50 (50:50)	62.0%	Exp: 54.7 ± 18.5; Cont: 58.6 ± 18.1^a^	≥18yrs; symptoms of ARI ≤4 d. Exclusion: no renal, hepatic, cardiac failure	Plasma cytokine/chemokine from Days 0 to 10	in‐hospital	0%
NSAID											
Hung et al^33^	Blinded, placebo‐controlled RCT	Hong Kong, 2014‐17	Seasonal [A/H3N2]	Hospital	120 (50:50)	58.3%	Exp: 70 (58.3‐38.3); Cont: 73.5 (60.3‐81.8)	≥18 y; symptoms of ILI ≤72 h; CrCl ≥ 30 mL/min; no CHF	Mortality	28‐day mortality	26.7%
NSAID and macrolide											
Hung et al^32^	Open‐label RCT	Hong Kong (1), 2015	Seasonal [A/H3N2]	Hospital	217 (49:51)	53.5%	Exp: 80 (72‐85); Cont: 81.5 (71‐87.3)	≥18 y; symptoms ≤72 h; infiltrate on CXR	Mortality	30‐day mortality	8.2%
mTOR inhibitors											
Wang et al^34^	Open‐label RCT	Taiwan (1), 2009‐11	Pandemic H1N1	ICU	38 (50:50)	78.9%	Exp: 46.7 ± 12.1; Cont: 51.5 ± 16.0 a	≥18 y; severe respiratory failure; ventilatory support	Not stated	In ICU	42.1%
Statin											
Chase et al^n^	Blinded, placebo‐controlled RCT	US (1), 2013‐18	Seasonal (A + B)	Hospital	116 (51:49)	37.9%	Exp: 34 (23‐51); Cont: 43 (29‐58)	≥18 y; prior statin therapy; liver cirrhosis/dysfunction	Change in Il‐6 after 72 h	In‐hospital	0%

Abbreviations: APACHE II, Acute Physiology, Age Chronic Health Evaluation II; ARI: Acute respiratory tract infection; CAP: community‐acquired pneumonia; CHF: congestive heart failure; COPD: chronic obstructive pulmonary disease; CrCl: creatinine clearance; CXR: chest radiograph; ICU: intensive care unit; ILI: influenza‐like illness; IQR: interquartile range; NEW: National Early Warning; NSAID: non‐steroidal anti‐inflammatory drug; RCT: randomised controlled trial.

a Mean ± standard deviation.

### 3.1 Passive immune therapy

Four randomised controlled trials—three double‐blind placebo‐controlled^25^ and one open‐label^28^—and one prospective cohort study 29 assessed the effect of passive immune therapy on mortality. A total of 693 participants were enrolled in these studies, of which 655 were analysed for the primary endpoint. A total of 323 received passive immune therapy (“experimental”) and 332 did not (“control”). Sixteen participants (Beigel et al^28^) were also included in the study by Davey et al. 26

All five studies administered a single infusion of a polyclonal passive immune therapy previously assessed to have a high titre to the influenza strain being treated. Hung et al^29^ administered convalescent plasma with a neutralising antibody titre (NAT) ≥1:160, which had been collected from patients who had recovered from pandemic A/H1N1‐2009 infection. Hung et al^25^ and Davey et al^26^ administered hyperimmune intravenous immunoglobulin (H‐IVIG) manufactured from convalescent plasma (Hung et al^25^ reported a NAT ≥1:160 to pandemic A/H1N1‐2009). Beigel et al^28^ and Beigel et al 27administered convalescent plasma from blood donation units with a haemagglutination inhibition (HAI) titre of at least 1:40 or 1:80 against the infecting influenza strain, respectively. Control groups received normal saline, standard IVIG or low‐titre anti‐influenza plasma depending on the study.

Only Beigel et al^28^ and Beigel et al 27enrolled children in addition to adults, but overall most study participants were adults (n = 631; 96.3%). On aggregate, study participants were evenly balanced by sex (male n = 341; 50.8%) and the median age of intervention groups ranged from 43 to 63 years. Mortality rates in the control groups were >20% in the older, smaller studies, and lower in the 3 larger, more recent RCTs (1.3%‐11.1%). In the cohort study (Hung et al^29^), recipients of immune therapy were more likely to present with cough and dyspnoea and were more likely to be obese. APACHE II and comorbidity scores were similar between groups. No significant difference between groups enrolled in the RCTs was noted. NAIs were administered as standard of care in all studies. Hung et al^29^ also reported the use of corticosteroids in 38 study participants.

**Figure 2:**
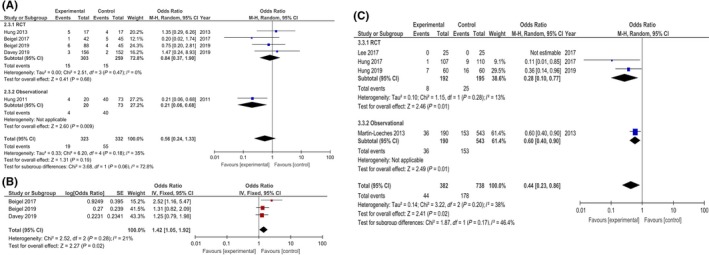
Forest Plots and Meta‐analysis of Adjunctive Immuno‐modulatory Therapy for Severe Influenza. A, Effect of passive immune therapy on crude mortality from RCTs and observational studies; B, Effect of passive immune therapy on clinical outcome at Day 7 using ordinal scale; C, Macrolide and/or NSAID effect on crude mortality from RCTs and observational studyCI: Confidence interval; M‐H: Mantel‐Haenszel; IV: generic inverse variance; NSAID: Non‐steroidal anti‐inflammatory drug; SE: Standard error

Two of the older published studies—an observational study and an RCT—reported similar point OR estimates of the effectiveness of immune therapy (Hung et al^29^ Beigel et al^28^). However, after including data from the two large newly published clinical trials no statistically significant evidence of an overall mortality benefit was identified from RCTs (OR = 0.84; 95% CI: 0.37‐1.90; *I* ^2^ = 0%; 562 participants) (Figure 2A).

Beigel et al^28^ reported the group who received passive immune therapy had significantly better clinical outcomes at Day 7 (*P* = .02) and after last hospital discharge (*P* = .029). This analysis was based on a 6‐point ordinal scale with outcomes scaled categorically from death, to ICU admission, to hospitalised with or without supplementary oxygen, to not hospitalised with or without resumption of normal activities. Using these clinical outcomes reported by Beigel et al^28^ we calculated a summary OR of 2.52 (95% CI: 1.16‐5.47; *P* = .019) using proportional odds logistic regression, where an OR >1.0 indicates a more favourable outcome with passive immune therapy.

On a similar ordinal scale to Beigel et al^28^ both Davey et al^26^and Beigel et al 27reported no significant difference in clinical outcomes with passive immune therapy at Day 7. Their ORs for the primary outcome of clinical status at Day 7 were 1.25 (95% CI: 0.79‐1.97; *P* = .33) and 1.22 (95% CI: 0.65‐2.29; *P* = .54), respectively. A post hoc meta‐analysis of Day 7 clinical outcomes from these 3 studies indicated overall significant clinical benefit from passive therapy (OR = 1.42; 95% CI: 1.05‐1.92; *P* = .02; *I* ^2^ = 21%; Figure 2B). Note the OR estimate from Davey 2019^26^used in this meta‐analysis excluded the 16 patients who were included from Beigel et al.^28^

In a subgroup analysis, Davey et al^26^ reported lower mortality at 7 days when H‐IVIG was administered to the 84 participants in this study with influenza B, though this difference was not statistically significant (0% vs 4.5% mortality). The summary OR for clinical outcome on the ordinal scale indicated clinical benefit with H‐IVIG for treatment of influenza B (OR = 3.19; 95% CI: 1.21‐8.42), but not influenza A (OR = 0.94; 95% CI: 0.55‐1.59).

Hung et al^25^ performed a subgroup analysis to explore the effect of the timing of passive immune therapy administration on outcomes, and reported that H‐IVIG administered within 5 days of symptoms onset was associated with significantly lower mortality in a multivariate model (OR = 0.14; 95% CI: 0.02‐0.92). However, this finding was based on a post hoc analysis and would imply 100% mortality when H‐IVIG is administered more than 5 days after symptom onset (5 out of 5 participants).

Hung et al^25^ reported that from similar baseline levels, viral load reduction was significantly faster in the group who received H‐IVIG. From a panel of 10 cytokine levels tested over the first 5 days of treatment, there were no significant differences in baseline levels, several were significantly higher on Day 1 after treatment in the group who received H‐IVIG ( IL‐10, IL‐1ra, MIP‐1α), while on Day 3 after treatment, several were significantly lower in the H‐IVIG group (IFN‐α2, TNF‐α). Influenza viral levels were not significantly different between intervention groups at Day 3 in the three studies which reported this outcome (Beigel et al,^28^ Davey et al,^26^ Beigel et al^27^).

In the study by Beigel et al,^28^ fewer serious adverse events (SAEs) occurred in plasma recipients than controls (20% vs 38%; *P* = .041). No transfusion‐associated side effects were described. The incidence of SAEs was similar in the studies from Beigel et al^27^and Davey et al.^26^ Hung et al^25^ did not systematically describe adverse events, but length of ICU and hospital stay were not significantly different between treatment groups.

### 3.2 Macrolide and NSAID therapy

Two studies investigated the effect of macrolide therapy alone: one prospective cohort study^30^ and one open‐label RCT^31^ (Table 1). A second open‐label RCT investigated the effect of a macrolide in combination with an NSAID^32^ for influenza treatment. A third RCT, blinded and placebo‐controlled investigated the effect of NSAID therapy alone. Data for this RCT were presented at European Congress of Clinical Microbiology & Infectious Diseases (ECCMID) 2019 and the Hong Kong Health Research Symposium 2019^33^(Hung et al). In total, 1120 participants were enrolled for these four studies, 322 received a macrolide (“experimental”) and 678 did not (“control”), while 167 received an NSAID (“experimental”) and 170 did not (“control”).

The cohort study enrolled adults admitted to the ICU with primary viral pneumonia and pandemic A/H1N1‐2009 influenza. Macrolides administered were clarithromycin (n = 99, 52.1%), azithromycin (n = 90, 47.4%) and erythromycin (n = 1, 0.5%). Experimental and control groups were similar in age and gender, but were more likely to be pregnant, immunosuppressed or have COPD. They were also more likely to receive adjunctive corticosteroid therapy, but had lower APACHE II scores and were less likely to have a haematological disease.

Lee et al^31^ and Hung et al^32^ enrolled adults hospitalised with seasonal influenza, and administered azithromycin 500 mg for 5 days, or clarithromycin 500 mg and naproxen 200 mg BD for 2 days to their experimental groups, respectively. Hung et al administered celecoxib 200 mg daily for 5 days or placebo to hospitalised adults. NAIs were administered as standard of care in all three studies.

There were no deaths recorded by Lee et al.^31^ The OR estimate derived from the two RCTs (Hung et al,^32^ Hung et al^33^) overlapped with the univariable OR from the observational study^30^ despite heterogeneity in study populations—for example, ICU and non‐ICU admissions and overall mortality rates of 4.6%‐25.7% (Figure 2C). Note after statistical adjustment in a multivariable logistic regression model, Martin‐Loeches et al^30^ reported no significant mortality benefit from macrolides (OR = 0.87; 95% CI: 0.55‐1.37; *P* = .5).

Subgroup analysis by Martin‐Loeches et al^30^ reported similar outcomes with macrolide administration in the subgroup of patients receiving mechanical ventilation compared to the whole cohort (aOR = 0.77; 95% CI: 0.44‐1.35). Length of ICU stay in survivors was also not significantly different between the group who received macrolides, and those who did not in the study by Martin‐Loeches et al.^30^

In the study by Hung et al,^32^ the group who received combination treatment with naproxen and clarithromycin were less likely to be admitted to a high dependency unit (*P* = .009) and had shorter hospital stays (*P* < .0001) compared to the group who received standard care. Virus titre and the number of patients with a proportion of NAI‐resistant quasispecies viruses >5% were also lower in the intervention group.

Lee et al^31^ reported no significant change in viral RNA decline or symptom resolution between treatment groups. However, in a generalised estimating model (GEE) adjusting for comorbidities and disease severity, the azithromycin treatment group had a significantly faster decline in the pro‐inflammatory cytokines CXCL9/MIG and IL‐17 from baseline to Day 10.

Hung et al^33^ reported a statistically significant reduction in serial IL‐6 and IL‐10 measurements from Day 1 to Day 5, and improvements in NEW score from Day 1 to Day 3 in the celecoxib group. There was a significantly lower incidence of ventilator‐associated pneumonia in patients receiving celecoxib (6.7% vs 13.3%; *P* = .04). Admission to ICU and length of hospitalisation was not significantly different between treatment groups, and no cardiac, renal or gastrointestinal side effects were reported.

### 3.3 mTOR inhibitor

One open‐label RCT studied the effect of the mTOR inhibitor sirolimus in the treatment of patients critically ill with influenza in ICU (Table 1).^34^ Thirty‐eight participants were enrolled in this study, 19 received 2mg sirolimus for 10 days (“experimental”), and 19 did not (“control”). All subjects also received corticosteroids. Three died (15.8%) in the experimental group compared to 8 (42.1%) in the control group. The effect of mTOR inhibitors on crude mortality from Wang et al^34^ was estimated with an OR = 0.26 (95% CI: 0.06‐1.19).

In the study by Wang et al,^34^ the organ dysfunction score (SOFA) improved faster in the sirolimus treatment group. Oxygenation also improved faster in this group, and a significantly shorter duration of ventilation was required. Among the 31/38 patients who had a repeat viral load on Day 7, a significantly larger proportion were undetectable in the sirolimus group (*P* < .05).

Wang et al^34^ described sirolimus‐associated adverse events over the 2‐week treatment (diarrhoea, elevated triglycerides and hyperglycaemia). It was not clear how the frequency of serious adverse events varied between treatment groups. Ventilator‐associated pneumonia occurred in three patients (15.8%) treated with sirolimus, and 6 (31.6%) in the control group (*P* = .45).

### 3.4 Statin

One unpublished blinded, placebo‐controlled RCT of adjunctive statin therapy was identified.^35^ Study investigators administered atorvastatin 40mg daily for 5‐7 days in patients hospitalised with influenza who were not receiving regular statins prior to hospital admission. Results were available from the study record at clinicaltrials.gov (Table 1).

No deaths were reported in this study in either treatment group. For the study primary endpoint, no significant difference between experimental and control groups in the change in IL‐6 levels from baseline to 72 hours was reported (*P* = .611). However, a significant improvement in symptom score over this time period was described in the atorvastatin treatment group (*P* = .029). Incidence of AEs was similar between treatment groups (6.8% vs. 8.8%), and no SAEs occurred.

### 3.5 Risk of bias assessment

The two observational studies were assessed to be of high quality.^29^, ^30^ The cohort study^29^ of passive immune therapy was downgraded by one point, as outcome assessment was adjusted for disease severity, but not propensity to receive passive immune therapy. The cohort study of macrolides therapy^30^ received the maximum nine points.

Overall, the five published RCTs were assessed at low risk of bias.^25^, ^28^, ^31^, ^32^, ^34^ While in four^28^, ^31^, ^32^, ^34^ of these RCTs participants and investigators were not blinded to group allocation and intervention received, it was judged unlikely this would have a significant effect on assessment of mortality. Complete assessment of the risk of bias was not possible for the four unpublished studies^33^, ^35^; however, all were reported as investigator and participant blinded placebo‐controlled RCTs and no bias concerns were evident from the available data.

### 3.6 GRADE assessment

GRADE assessments of the certainty of evidence were conducted based on summary effect data from RCTs only. Certainty of evidence was graded as low for passive immune therapy, due to imprecision and uncertainty surrounding the applicability of evidence in individuals with severe influenza at highest risk of mortality and imprecision. The two largest clinical trials (Davey et al,^26^ Beigel et al^27^) were not powered to detect mortality difference. Certainty of evidence for other adjunctive therapies was also graded as low reflecting variability in interventions and/or imprecision (Table 2 **)**.

**Table 2:** Immunomodulatory therapy for influenza infection. Patient or population: severe influenza; Setting: in hospital; Intervention: immunomodulatory therapy; Comparison: no immunomodulatory therapy

Intervention	Outcomes	No. of participants (studies) Follow‐up	Certainty of the evidence (GRADE)	Relative effect (95% CI)	Anticipated absolute effects	
					Risk without immunomodulatory therapy^a^	Risk difference with immunomodulatory therapy
Passive immune therapy	Mortality (RCTs only)	562 (4 RCTs)	⨁⨁◯◯ Low^b^	OR 0.84 (0.37 to 1.90)	58 per 1000	9 fewer per 1000 (36 fewer to 47 more)
Macrolides or NSAIDs	Mortality (RCT)	387 (3 RCTs)	⨁⨁◯◯ Low^c^	OR 0.28 (0.10 to 0.77)	128 per 1000	89 fewer per 1000 (114 fewer to 26 fewer)
mTOR inhibitors	Mortality (RCT)	38 (1 RCT)	⨁⨁◯◯ LOW^d^	OR 0.26 (0.06 to 1.19)	421 per 1000	262 fewer per 1000 (379 fewer to 43 more)
Statins	Mortality (RCT)	116 (1 RCT)	Not Assessable^e^	–	–	–

Abbreviations: CI: confidence interval; NSAID: non‐steroidal anti‐inflammatory drug; OR: odds ratio; RCT: randomised controlled trial.

a The risk in the intervention group (and its 95% confidence interval) is based on the assumed risk in the comparison group and the relative effect of the intervention (and its 95% CI).

b Certainty downgraded by two for imprecision and indirectness due to differences between study populations.

c Certainty downgraded by two for indirectness due to differences in interventions and populations.

d Certainty downgraded by two for imprecision due to the small number of study participants.

e No mortality in the clinical trial evaluated.

### 3.7 Ongoing studies

One RCT of passive immune therapy, which has been reported as completed, was identified from the screen of clinical trial databases, but results are not expected till 2020 (Appendix ). One additional RCT of the macrolide clarithromycin was also identified but at the time of review had not yet been initiated.

## 4 DISCUSSION

The systematic review identified eleven completed studies of non‐corticosteroid immunomodulatory therapies, including some high‐quality RCTs. The results of these studies indicate there is unlikely to be a substantial mortality benefit from passive immune therapy as an adjunct to conventional antiviral therapy for the treatment of severe seasonal influenza. Currently, there is insufficient evidence to recommend routine administration of any of the reviewed adjunctive therapies; however, the results from study of macrolides and NSAIDs warrant further study in well‐designed RCTs.

The highest quality evidence uncovered through this systematic review was for the effect of passive immune therapy on severe influenza mortality. Our pooled estimate after including two recently completed and relatively large RCTs indicated it is unlikely to be of benefit in reducing mortality in most situations. This conclusion is different from previous reviews of passive immune therapy, but exclusion of non‐randomised and uncontrolled historical studies conducted during the 1918 pandemic suggests our summary effect estimate is more likely relevant to treatment of influenza today. One further study of passive immune therapy is expected to report results within the next year. This is a phase 2 dose‐ranging study with pharmacologic and safety primary endpoints, which recruited 65 individuals, and its results are unlikely to substantially alter the conclusions of this review.

There was statistically significant evidence of clinical benefit from passive immune therapy in the post hoc meta‐analysis of Day 7 clinical outcomes. Outcomes in this analysis are ordered on an 6‐point categorical scale from death to “not hospitalised with resumption of normal activities.” This endpoint has been adopted for a number of ongoing phase III clinical trials of influenza antivirals,^36^, ^37^ reflecting uncertainty as to what is the most appropriate single endpoint for severe influenza and 2011 guidance for industry from the US FDA.^38^

The summary OR from the meta‐analysis indicates the magnitude of any benefit is likely to be small, while the relevance of desirable but less clinically significant endpoints in this scale is questionable for passive immune therapy. The cost and difficulty of obtaining significant volumes of convalescent plasma/serum currently constrain its accessibility as part of routine care for severe seasonal influenza. Further, as highlighted by Beigel et al^27^the apparent requirement for antibodies with high‐binding affinity to the specific infecting influenza strain is also problematic. Passive immune therapy may, however, retain a role to reduce the substantial morbidity and mortality from highly pathogenic avian influenza such as A/H7N9 and A/H5N1, and other emerging severe acute respiratory viruses such as MERS Co‐V, or in the event of a new influenza pandemic.

In terms of other immunomodulatory agents, despite the apparent mortality benefit with adjunctive mTOR inhibitors observed in the small RCT by Wang et al^34^, subsequent studies have questioned this finding. For example, in an animal study, rapamycin attenuated antigen‐specific immune responses without reducing pulmonary inflammation, but impairing viral clearance.^39^ The clinical use of powerful immunosuppressants, including mTOR inhibitors and corticosteroids, needs to be carefully judged to determine whether benefits outweigh risks. Which populations are most likely to benefit, and the optimal timing of administration (eg before or after the onset of critical illness) are important factors that are not well understood.

The immunomodulatory effects of statins have been well described, and this may be the mechanism of action for some of their observed cardiovascular benefits.^40^ The immune modulation in long‐term use has been associated with reduced influenza vaccine effectiveness^41^ though not consistently.^42^ Conversely, influenza infection in people who are receiving statins has been reported to be at lower risk of hospitalisation or death in a large observational study.^43^ The RCT by Chase 2019^35^ suggested some benefit on clinical symptoms with initiation of statin therapy but was not powered to detect any mortality benefit. Of note, an RCT of rosuvastatin for sepsis‐associated ARDS was halted for futility and may have contributed to hepatic and renal dysfunction.^44^ It is plausible that the time course for the onset of any immunomodulator effect precludes any benefit when initiated during acute infection.

Finally, what is the role of macrolides and NSAIDs in the management of severe influenza? Both are frequently administered, either because of diagnostic uncertainty as to whether there is a concomitant bacterial infection or for symptom relief. The effects of these agents are complex, involving multiple inflammatory pathways. For example, changes in the respiratory tract microbiome from macrolide antibiotics could affect susceptibility to secondary bacterial infection or the inflammatory response itself,^45^ and animal studies have suggested that selective inhibition of COX‐2 may be the most effective due to its inducible, pro‐inflammatory nature.^46^

As low‐cost therapies with a favourable adverse effect profile, these are both attractive adjunctive therapies. The significant reduction in mortality observed in the two RCTs published by Hung et al^25^, ^32^ is encouraging, but these studies were conducted in a relatively select population (A/H3N2, symptoms for <72 hours, older population) with high mortality rates compared with the other studies of hospitalised patients in this review. Further study is clearly necessary before they can be recommended as part of standard of care, and to tease out which (if any) agent, dose or timing of administration, and population are the most effective.

This systematic review has several limitations. Focusing on the treatment of influenza in hospitalised patients, and the effects of immunomodulatory therapy on mortality overlooks their potential benefit/harm in the treatment of milder infections. The design of the review did not allow us to systematically assess published evidence as to whether immunomodulatory therapy may be able to reduce hospital admissions, speed symptom recovery or prevent influenza‐associated complications. Additional limitations include not searching for grey literature and the small number of abstracts with potentially eligible studies for inclusion, but where we were unable to locate full‐text copies of articles. We also included data from a number of unpublished studies, and it is possible published results may change—though this is unlikely for mortality.

The decision to exclude observational studies, which did not perform multivariate adjustment for confounding, is not likely to alter the overall conclusions of our review, as the majority of excluded studies that were included in other systematic reviews were of small size. Incorporating these studies into the review would ideally require an individual patient data meta‐analysis.

The influenza treatment landscape may also change significantly over the coming years, if any of the antivirals currently in clinical development demonstrate superiority to current standard of care with NAI. If so, a re‐assessment of the role of immunomodulatory therapy will be required.

There is insufficient evidence currently for any of the assessed immunomodulatory therapies to be routinely used in clinical practice for the treatment of influenza infection. The results of studies of macrolides, NSAIDs and mTOR inhibitors are encouraging, however, and warrant further study in RCTs.
