# Supplementary material for: Passive immune therapy and other immunomodulatory agents for the treatment of severe influenza: Systematic review and meta‐analysis
Source: Influenza Other Respir Viruses. 2019 Nov 16;14(2):226–36. doi: 10.1111/irv.12699 (PMC7040980; doi:10.1111/irv.12699)
Supplement: Supplementary file 1 [file IRV-14-226-s001.docx]

**Appendix 1 :Medline Search Construct and Inclusion and Exclusion Criteria**

**Search methods for identification of studies**

**Electronic searches**

We will search the following electronic databases: Cochrane Central Register of Controlled Trials (CENTRAL), MEDLINE and EMBASE. There will be no restriction on date or language of publication.

The following core search strategy will be implemented in MEDLINE and adapted for other databases as necessary.

1 Influenza, Human/

2 exp Influenzavirus A/

3 exp Influenzavirus B/

4 (influenza* or flu).ti.

5 (h1n1 or h3n2 or h5n1 or h7n9).mp.

6 or/1-5

This core database will then be searched separately with terms appropriate for each of the identified adjunctive therapies.

1. Corticosteroids (adapted from ^1^)

7 (corticoid* or corticosteroid* or dexamethasone* or glucocorticoid* or hydrocortisone* or methylprednisolone* or prednisolone* or prednisone* or steroid*).mp.

8 6 and 7

1. Passive immune therapy (adapted from ^2^)

9 (convales* and (plasma or sera or (blood and product*) or serum) or hyperimmune immunoglobulin or hyper-immun* or hyperimmun* or serotherapy).mp.

10 6 and 9

1. Statins (adapted from ^3^

11 (atorvastatin or cerivastatin or dalvastatin or fluindostatin or fluvastatin or HMG-CoA* or Hydroxymethylglutaryl* or lovastatin or medostatin or pitavastatin or pravastatin or rosuvastatin or simvastatin or statin*).mp.

12 6 and 11

1. Macrolides (adapted from ^4^)

13 (azithromycin or clarithromycin or erythromycin or josamycin or macrolide* or midecamycin or oleandomycin or roxithromycin or solithromycin or spiramycin or telithromycin or troleandomycin).mp.

14 6 and 13

1. mTOR inhibitors

15 (everolimus or sirolimus or temisirolimus or rapamycin).mp.

16 6 and 15

1. NSAIDs (adapted from ^5^)

17 (aspirin or celecoxib or diclofenac or dipyrone or flurbiprofen, or ibuprofen, or indomet?acin or ketoprofen or ketorolac or mefenamic acid or naproxen or nefopam or nimesulide or phenylbutazone or piroxicam).mp.

18 6 and 17

### **Searching other resources**

### Clinicaltrials.gov and the WHOs International Clinical Trials Registry Platform (ICTRP) was also searched for ongoing clinical trials. Web of Science was used for citation searching by collating the bibliographies and citations of included studies, to identify additional studies which may be eligible.

**Criteria for considering studies**

| **Inclusion Criteria** |
| --- |
| Microbiologically confirmed influenza infection, for example by PCR, rapid antigen test or culture |
| Influenza of any type/subtype: seasonal (A/H1, A/H3, B), pandemic, or avian/animal influenza (e.g. A/H5, A/H7) |
| Study participants admitted to hospital, but no restrictions on setting (e.g. ICU or general ward) |
| No restrictions on demographics of study participants (e.g. age, sex, geographic location, co-morbidity, pregnancy) |
| No restrictions on dose, route of administration or duration of adjunctive therapy |
| No restriction on timing of adjunctive therapy after hospital/ICU admission or symptom onset |
| Studies of combinations of adjunctive therapy will be included |
| Study reporting at least one of the primary or secondary outcomes |
| No date or language restrictions |
| Study published in peer-reviewed literature |
| **Exclusion Criteria** |
| Studies which include <10 participants |
| Studies describing prophylactic interventions |
| Studies conducted in animals |
| Observational studies which do not attempt to adjust outcome measures for confounding (for example differences in disease severity between experimental and control group) |

*Table 1: Summary of study inclusion and exclusion criteria*

**References:**

1. Rodrigo C, Leonardi-Bee J, Nguyen-Van-Tam J, Lim WS. Corticosteroids as adjunctive therapy in the treatment of influenza. *Cochrane Database Syst Rev*. 2016;3:CD010406. doi:10.1002/14651858.CD010406.pub2

2. Mair-Jenkins J, Saavedra-Campos M, Baillie JK, et al. The effectiveness of convalescent plasma and hyperimmune immunoglobulin for the treatment of severe acute respiratory infections of viral etiology: a systematic review and exploratory meta-analysis. *J Infect Dis*. 2015;211(1):80-90. doi:10.1093/infdis/jiu396

3. Taylor F, Huffman MD, Macedo AF, et al. Statins for the primary prevention of cardiovascular disease. *Cochrane Database Syst Rev*. 2013;(1):CD004816. doi:10.1002/14651858.CD004816.pub5

4. Kelly C, Chalmers JD, Crossingham I, et al. Macrolide antibiotics for bronchiectasis. *Cochrane Database Syst Rev*. 2018;3:CD012406. doi:10.1002/14651858.CD012406.pub2

5. Cooper TE, Heathcote LC, Anderson B, Grégoire M-C, Ljungman G, Eccleston C. Non-steroidal anti-inflammatory drugs (NSAIDs) for cancer-related pain in children and adolescents. *Cochrane Database Syst Rev*. 2017;7:CD012563. doi:10.1002/14651858.CD012563.pub2
