# Supplementary material for: Passive immune therapy and other immunomodulatory agents for the treatment of severe influenza: Systematic review and meta‐analysis
Source: Influenza Other Respir Viruses. 2019 Nov 16;14(2):226–36. doi: 10.1111/irv.12699 (PMC7040980; doi:10.1111/irv.12699)
Supplement: Supplementary file 3 [file IRV-14-226-s003.doc]

Group: Paper:

| **Assessment of quality of a cohort study – Newcastle Ottawa Scale** |  |
| --- | --- |
| **Selection** (tick one box in each section) |  |
| 1. Representativeness of the intervention cohorta) truly representative of the average hospitalised individual with influenza infection ★b) somewhat representative of the average hospitalised individual with influenza infection ★c) selected group of patients, e.g. only certain socio-economic groups/areasd) no description of the derivation of the cohort | 🞏  🞏  🞏  🞏 |
| 2. Selection of the non intervention cohorta) drawn from the same community as the intervention cohort ★b) drawn from a different sourcec) no description of the derivation of the non intervention cohort | 🞏  🞏  🞏 |
| 3. Ascertainment of interventiona) secure record (eg clinical notes) ★b) structured interview ★c) written self reportd) other / no description | 🞏  🞏  🞏  🞏 |
| 4. Demonstration that outcome of interest was not present at start of studya) yes ★b) no | 🞏  🞏 |
| **Comparability** (tick one or both boxes, as appropriate) |  |
| 1. Comparability of cohorts on the basis of the design or analysisa) study controls for infection severity ★b) study controls for propensity to receive the intervention ★ | 🞏  🞏 |
| **Outcome** (tick one box in each section) |  |
| 1. Assessment of outcomea) independent blind assessment ★b) record linkage ★c) self report d) other / no description | 🞏  🞏  🞏  🞏 |
| 2. Was follow up long enough for outcomes to occura) yes, if duration of follow-up was at least that hospital admission ★b) no, if duration of follow-up is only within ICU or is not known | 🞏  🞏 |
| 3. Adequacy of follow up of cohortsa) complete follow up: all subjects accounted for ★b) subjects lost to follow up unlikely to introduce bias: number lost <= 5%, ★ or description of those lost suggesting no different from those followedc) follow up rate < 95% and no description of those lostd) no statement | 🞏  🞏  🞏  🞏 |

NB Underlined text ‘customised’ for the intervention being reviewed

**NOS – CODING MANUAL FOR COHORT STUDIES**

***SELECTION***

1. **Representativeness of the Exposed Cohort (NB exposure = intervention)**

Item is assessing the representativeness of exposed individuals who have been hospitalised, not the representativeness of the study sample from some general population. For example, subjects derived from groups likely to contain exposed people are likely to be representative of exposed individuals, while they are not representative of all people the community.

*Allocation of stars as per rating sheet*

1. **Selection of the Non-Exposed Cohort**

*Allocation of stars as per rating sheet*

1. **Ascertainment of Exposure**

*Allocation of stars as per rating sheet*

1. **Demonstration That Outcome of Interest Was Not Present at Start of Study**

In the case of mortality studies, outcome of interest is still the presence of a disease/incident, rather than death. That is to say that a statement of no history of disease or incident earns a star.

***COMPARABILITY***

1. **Comparability of Cohorts on the Basis of the Design or Analysis**

Either exposed and non-exposed individuals must be matched in the design and/or confounders must be adjusted for in the analysis. Statements of no differences between groups or that differences were not statistically significant are not sufficient for establishing comparability. Note: If the relative risk for the exposure of interest is adjusted for the confounders listed, then the groups will be considered to be comparable on each variable used in the adjustment.

*A maximum of 2 stars can be allotted in this category.*

***OUTCOME***

1. **Assessment of Outcome**

For some outcomes, reference to the medical record is sufficient to satisfy the requirement for confirmation. This may not be adequate for other outcomes where reference to specific tests or measures would be required.

1. Independent or blind assessment stated in the paper, or confirmation of the outcome by reference to secure records (health records, etc.)
2. Record linkage (e.g. identified through ICD codes on database records)
3. Self-report (i.e. no reference to original health records or documented source to confirm the outcome)
4. No description.
5. **Was Follow-Up Long Enough for Outcomes to Occur**

An acceptable length of time should be decided before quality assessment begins.

1. **Adequacy of Follow Up of Cohorts**

This item assesses the follow-up of the exposed and non-exposed cohorts to ensure that losses are not related to either the exposure or the outcome.

*Allocation of stars as per rating sheet*
