# Supplementary material for: Passive immune therapy and other immunomodulatory agents for the treatment of severe influenza: Systematic review and meta‐analysis
Source: Influenza Other Respir Viruses. 2019 Nov 16;14(2):226–36. doi: 10.1111/irv.12699 (PMC7040980; doi:10.1111/irv.12699)
Supplement: Supplementary file 4 [file IRV-14-226-s004.docx]

#### **Appendix 4: Summary of ongoing studies identified from clinical trial databases**

| Reference | Intervention | Study design | Participants | Primary outcome | Status |
| --- | --- | --- | --- | --- | --- |
| NCT03315104 ^1^ | Hyperimmune IVIG: high-dose vs. low-dose vs. placebo | Phase II blinded RCT | Hospitalised adults with influenza A | Safety and pharmacokinetics | Recruiting (estimated completion June 2019) |
| NCT03824847 ^2^ | Clarithromycin vs. placebo | Phase IV blinded RCT | Adults with influenza | Symptom resolution and viral shedding | Not yet recruiting |

**References**

1. Exploring Safety & Clinical Benefit of Anti-Influenza Immunoglobulin Intravenous in Hospitalized Adults With Influenza A - Full Text View - ClinicalTrials.gov. https://clinicaltrials.gov/ct2/show/NCT03315104. Accessed February 14, 2019.

2. Immunomodulatory Effect of Macrolides in Naturally Occurring Influenza Virus Infections in the Community - Full Text View - ClinicalTrials.gov. https://clinicaltrials.gov/ct2/show/NCT03824847. Accessed February 14, 2019.
